# Stakeholder alliances are essential to reduce the scourge of plastic pollution

**DOI:** 10.1038/s41467-023-38613-3

**Published:** 2023-05-22

**Authors:** Richard S. Lampitt, Stephen Fletcher, Matthew Cole, Alice Kloker, Stefan Krause, Fran O’Hara, Peter Ryde, Mahua Saha, Anastasia Voronkova, Adrian Whyle

**Affiliations:** 1 National Oceanography Centre, European Way, SO14 3ZH Southampton, UK; 2 School of the Environment, Geography, and Geosciences, University of Portsmouth, Portsmouth, PO1 2UP UK; 3 Plymouth Marine Laboratory, Prospect Place, Plymouth, PL1 3DH UK; 4 School of Geography, Earth and Environmental Science, University of Birmingham, Birmingham, B15 2TT UK; 5 Univ Lyon, Université Lyon 1 Claude Bernard, LEHNA, 3 Rue M. Audin, 69518 Vaulx-en-Velin, Cedex France; 6 Scarlet Design Int. Ltd., 3 Thistle Way, Llandaff, Cardiff, CF5 2BU UK; 7 CSIR-National Institute of Oceanography, Dona Paula, Goa, 403 004 India; 8 School of Psychology, University of Plymouth, Plymouth, Pl4 8AA UK

**Keywords:** Environmental impact, Decision making, Risk factors

## Abstract

Progress to reduce plastic pollution has been painfully slow and the consequent damage to the natural environment and to human health is likely to increase further. This has been because the views and ways of working of four distinct stakeholder communities are not sufficiently well integrated. (1) Scientists, (2) industry, (3) society at large and (4) those making policy and legislation must in future find ways to work together.

About 400 million tonnes of plastic waste are generated every year, and this quantity is expected to increase dramatically over the coming decades^1^. Less than 20% of this waste is managed adequately, with most of it being incinerated, discarded into landfill or released into the natural environment. Much of the public concern has been focussed on the effects of plastic pollution on marine environments, but there is also increasing evidence of the deleterious impacts of plastics in terrestrial and freshwater ecosystems. According to a recent report by the UN Environment Programme (UNEP), over 10 million metric tonnes of plastic enter aquatic ecosystems annually with evidence of significant damage to ecosystem structure and function^2,3^. In contrast to public perception, the majority of the plastic currently in the sea is not floating but is below the surface and exists as tiny, microscopic particles^4^ and thus impossible to remove. Furthermore, once out of the direct action of waves and sunlight, degradation is extremely slow;^5^ although the processes driving this are yet to be understood. Whilst clean-up operations can reduce exposure to large items of plastic in localised pollution hotspots for short periods of time, the only sustainable way to reduce plastic pollution at a global scale is to radically reduce plastic entering the natural environment in the first place. Concern about the impacts of plastic pollution on human health is growing and there is increasing evidence of harm, largely owing to the presence of additives, such as certain plasticizers, fire retardants and colouring agents, some of which act as endocrine disrupting substances and carcinogens^6^. An often-overlooked fact is that harm to human health from mismanaged plastic waste disproportionately impacts people living in poorer countries^7^.

## Stakeholders in tackling plastic pollution

Given the international nature of the plastics supply chain, any action to tackle plastic pollution must be coordinated globally and across all main actors. It is our view that, in order to be effective, a comprehensive approach to tackle plastic pollution must involve the input and support of a wide and diverse groups of interdisciplinary stakeholders. We identify four different relevant communities of stakeholders, each of which must be included and given the opportunity to contribute to solutions to this crisis. The four major communities comprise;

1. Scientific
2. Industrial
3. Societal
4. Policy makers

Each of these stakeholders have very different characteristics, perspectives, ways of working and responses to the global plastic pollution challenge. We strongly believe that all these communities must be equitable partners of any international treaty, assembled in a “Global Evidence Community” focussed on reducing harms from plastics to human and ecosystem health.

Such a “community” will have the very specific role of bringing together diverse stakeholder communities into an effective and representative body of knowledge creators, solution providers and advocates of sustainable change. These different communities have been identified previously as being the essential players^2^ but progress to integrate the diverse experiences and perspectives they can provide has largely been slow and ineffective. The specific key challenges facing these four communities are:

### Scientific

The scientific community has the role of capturing insights from across all disciplines that generate actionable research data. To target solutions there is a need for a much better understanding about the extent to which specific sources produce or release plastic, the mechanisms by which it reaches the natural environment, how and at what speed it is transformed over time and what is its eventual fate. In addition, the toxicity of the plastic waste materials (including the synthetic polymers as well as additives and adsorbed co-pollutants) at current and predicted future concentrations must be better understood from the perspective of ecosystem function and human health^8,9^. The World Health Organisation evaluated available data to assess the risk to human health but were unable to complete a risk assessment because data, sampling and experimental designs were inconsistent and the methods for interpretation of toxicological studies were unclear^10^. Nevertheless, there is increasing evidence of harms of various types^3,11,12^.

It is essential to address plastics pollution (sources, toxicology and risk assessment) in the same way as is being done for greenhouse gas emissions, climate change and biodiversity loss. To achieve this, the research outlined above is required and most importantly all of this research must be in the context of a global threat characterised by large regional and local variability. This will require targeted policy-relevant research with focused funding calls, and we call on national scientific and business leaders to be the torchbearers in identifying the spectrum of scientific research, which is essential if evidence-based solutions are to be found.

### Industrial

For the chemical, manufacturing and agrochemical industry sectors, a transformation towards more sustainable plastic use and end of life solutions is needed. This must take place across the spectrum of activities from production of feedstocks for polymer production, manufacture of finished articles and their recycling at end of life. We believe that industry must assume a high level of responsibility for providing accessible, resource efficient waste management solutions and that they have the flexibility and ingenuity to achieve this in response to public concern, legislation, economic realities and the need for material security. Measures such as Extended Producer Responsibility (EPR) and Deposit Return Systems (DRS) play an essential role and are currently being implemented in Europe but need to be more broadly adopted across the globe.

The need for action has become compelling and this transformation needs to be accelerated immediately. There are a number of areas in which progress is urgently required and industry is well aware of these and have started to address them. However, while encouraging, such actions need to increase in scope and greatly accelerate. These include the replacement of components and additives which are known to be harmful by ones which are environmentally benign, a move to non-fossil-based feedstocks, reduced consumption and increased circularity.

The design of environmentally degradable polymers requires not only a rethinking of expectations on plastic persistence and longevity, but also a definition of industry standards for biodegradable plastics. This will ensure that there is a clear understanding of how to dispose of such plastics in a responsible manner at their end of life through, for example, food waste collection and preventing them from contaminating those plastics streams collected for recycling. Biodegradable plastics are not however a solution to the littering behaviour of society. The substantial levels of creativity and determination which characterises the industrial sector can lead to the necessary new products, feedstocks, systems, and processes as long as the incentives are fully developed. It is critical that all private sector action falls under a legal obligation for the transparent disclosure of progress against pre-agreed targets.

### Societal

It is not possible to generalise about “society” as a stakeholder, but we use the term here to distinguish those who are influenced by plastic (the entire human race) from those who are part of scientific, industrial or decision-making communities. Many social groups and societies have considerable concern about plastic pollution^13^ although they may struggle to change their behaviours due to external circumstances^14^. In contrast, some societies do not consider plastic pollution to be a serious hazard^15^ in comparison to the other challenges of inadequate nutrition, health and housing which pose existential threats to survival. Social class and caste, access to secure income and geography are all variables which impact not only perception of the problem of plastic pollution but the consequences of mismanaged plastic waste and impacts upon health.

We believe it is important not to slide into stimulating eco-anxiety and fear^16^. Putting all the weight of sustainable behaviours on “public members” is unrealistic, as in many cases those behaviours will be limited by barriers such as the lack of affordable sustainable product alternatives or accessible, affordable and reliable recycling system. Instead, we believe the underlying challenge to be the empowerment of people to make informed decisions based on transparent information (such as clearer labelling often misunderstood by consumers^17^, reducing barriers to sustainability, and cultivating new social norms for consumption and disposal^18^). While a global framework is crucial for high-level accountability, local context and localised solutions must always be at the forefront of decision-making.

### Policy makers

The fourth community comprises those who develop policy and ultimately legislation which can have tangible and positive consequences if and when supported by public and industrial sectors. Decision-makers at local, regional, national and global/international levels are all included in this category. There are several major challenges in the development of effective policy, with many similarities to those addressed by the Intergovernmental Panel on Climate Change (IPCC).

For plastic pollution, major challenges are the definition of standards and thresholds of harm and the coordination of efforts across regions. Plastic pollution is a global, transboundary problem that causes local consequences.

In March 2022, nearly 200 nations endorsed a resolution at the UN Environment Assembly (UNEA) in Nairobi to end plastic pollution using a global legally binding agreement or treaty to be developed by the end of 2024^19,20^. This UN-led process is a major opportunity to integrate the stakeholder communities so that their diverse perspectives are heard, understood, and acted upon. There is an urgent need to reduce plastic entering the supply chain, to ensure that this plastic is safe and is circulated as much as possible (potentially through the widespread introduction of extended producer responsibility schemes, requirements for reusable packaging, and more effective waste collection and recycling), and that there are plans to deal with both legacy waste and existing pollution. It is only when the holistic approach we propose has been adopted that practical, long-lasting, enforceable, and effective actions can be developed and enacted at local, regional, national and global levels.

## Integration of communities is essential

We propose that the Global Evidence Community organised around reducing harm caused by plastics will be drawn from the four stakeholder communities outlined above and will have the specific role to interact vigorously and positively through a variety of mechanisms including social media, publicity, conferences, and secondments to ensure the diversity of evidence available is brought to the negotiating table. It will be important to engage with other groups, including the High Ambition Coalition of Countries to End Plastic Pollution, the Scientists Coalition for an Effective Plastics Treaty, the Business Coalition for a Global Plastics Treaty, and the UNEP-backed independent intergovernmental science-policy panel to contribute to the sound management of chemicals and waste and to prevent pollution. A crucial feature of these initiatives which is often of lower priority is the understanding of the diversity of societal perspectives and opportunities for change. In our view this fourth and extremely complex component of the global evidence community must be properly represented.

None of these communities have all the expertise needed to address this serious and urgent issue to reduce the scourge of plastic pollution but all have a crucial role in finding a solution. By engaging with the proposed interdisciplinary stakeholders such incentives will become extremely obvious and compelling. We therefore call on the international community and UNEP to demonstrate leadership, courage and integrity to work collaboratively to create the new paradigms required to end the pollution of our environment and safeguard the health of our planet. The UNEP meeting in Paris in May/June 2023 building on the March 2022 UNEA meeting is an important opportunity to develop these objectives still further.
